# Physicochemical Characterization of Woody Lignocellulosic Biomass and Charcoal for Bio-energy Heat Generation

**DOI:** 10.1038/s41598-023-46054-7

**Published:** 2023-11-07

**Authors:** Adebayo S. Olabisi, Ayokunle O. Balogun, Taiwo O. Oni, Bamidele S. Fakinle, Farid Sotoudehnia, Armando G. McDonald, Peter P. Ikubanni

**Affiliations:** 1 Department of Mechanical Engineering, College of Engineering, Williams Tubman university, Maryland County, Harper, Liberia; 2 Department of Mechanical Engineering, College of Engineering, Landmark University, Omu-Aran, Nigeria; 3 Department of Chemical Engineering, College of Engineering, Landmark University, Omu-Aran, Nigeria; 4 Department of Forest, Rangeland and Fire Science, University of Idaho, Moscow, ID 83844-1132 USA

**Keywords:** Energy science and technology, Engineering

## Abstract

Biomass and its interactions for heat generation have received little attention. In this study, the woody biomass materials were *Prosopis africana* (PA), *Harungana madascariences* (HM), *Vitrllaria paradoxa* (VP), and *Afzelia africana* (AA). The composition (extractives, carbohydrate, and lignin) of the biomass was determined. The biomass was converted to charcoal in a traditional kiln. A thermo-kinetic examination of the charcoal samples was carried out. The kinetic parameters and potential reaction mechanisms involved in the decomposition process were both obtained using the integral (Flynn–Wall Ozawa) isoconversional methods in conjunction with the Coats-Redfern approach. The activation energy profiles for the charcoal samples in oxidizing atmospheres were 548 kJ/mol for AA, 274 kJ/mol for VP, 548 kJ/mol for PA, and 274 kJ/mol for HM. All charcoal samples underwent comprehensive, multi-step, complex reaction pathways for thermal degradation. The charcoal samples exhibit not only great potential for biochemical extraction but also for bioenergy applications. The significant amount of combustion characteristics in the raw biomass and charcoal samples indicates that each type of wood charcoal produced has more fixed carbon, less ash, and less volatile matter, all of which are desirable for the thermo-chemical conversion of biomass for the production of heat.

## Introduction

A country's ability to industrialize, develop, and flourish economically depends on its access to energy. Due to environmental concerns and the limited resources associated with burning fossil fuels, there is a lot of interest in alternative and sustainable energy sources^1^. Charcoal is a traditional energy source that is still widely used, primarily in developing countries. Charcoal is a solid residue made from tree offcuts and farm clearing site debris that is burned at temperatures > 300 °C in a confined space with constrained air flow. In light of the large amount of organic substance and sufficiently rich calorific value of the charcoal after cooling, it is one of the viable alternatives for an alternative fuel comparable to coal^2^. Charcoal can be produced from biomass, such as tree offcuts and partially decomposed plant waste, to replace briquettes, firewood, gas, coal, electricity, and other sources of domestic and industrial energy.

Since alternative energy sources, such as electricity, gas, and kerosene, are either in short supply or dreadfully inadequate when they do exist and are out of the reach of most people, fuel wood has become the principal energy source for distant settlements and even large eating establishments in modern times. In comparison to briquettes and firewood, charcoal burns more cleanly in cook stoves depending on the kind of tree offcut used to make it. Thus, the development of charcoal from tree offcuts transforms waste wood into a fuel source that can replace non-renewable energy sources.

In order to obtain beneficial energy sources from tree offcuts and waste, thermal technologies can be used in three different ways, such as pyrolysis, gasification, and combustion^3^. Biomass is a renewable energy source that significantly reduces CO_2_ emissions and produces a stable flame during the combustion process^3^. So, burning biomass and charcoal could increase overall combustion efficiency. Combustion currently accounts for more than 97% of all bioenergy produced globally, making it the easiest and most straightforward technology conversion for using biomass^4^. In order to provide sustainable supplies of biomass, this study investigates the use of energy crops and native wastes. Additionally, there are a lot of untapped biomass remnants that can be used as a resource for sustenance^5^. The amount of wood waste produced state wide (about 2000 sawmills) is estimated to be 104,000 m^3^ per day, yet there is very little knowledge about Nigerian energy crops and woodland resources^6^.

Thermogravimetric analysis (TGA), which has a variety of uses for research and technical evaluation of fuels, is frequently used to study combustion characteristics^7^. TGA, which measures the kinetic characteristics of fuel combustion, is thought to be more efficient and advantageous. The four main stages of combustion, including fixed carbon (FC) combustion, oxidation, volatile ignition, and moisture evaporation, may all be detected by TGA, making it a very helpful instrument for studying fuel devolatilization and combustion from a kinetic perspective^8,9^. The combustion of charcoal and its interactions, however, have received little attention. First, this makes it challenging to compare the effectiveness of the potential replacement and the current fuel source using empirical evidence. Second, without the necessary technical information, modelling the accompanying heat transfer processes and designing an optimal system are quite challenging. The commercially available wood used for charcoal in Nigeria must therefore be subjected to thorough chemical, thermal and structural analyses to determine its composition, combustion parameters, and kinetic information.

In this work, four Nigerian tree species were studied and converted to charcoal in an earth mound kiln. The wood biomass samples were characterized for chemical composition and proximate analysis. TGA was used to analyze the combustion properties of charcoal and woody biomass at three heating rates (5, 10, and 20 °C/min) in air.

The woody lignocellulosic biomass and charcoal can be used to produce heat or power utilizing machinery like furnaces, boilers, steam turbines, and turbo-generators and formation of the related thermochemical techniques as well as the design and optimization of reactors/combustors would benefit greatly from the kinetic data.

## Methodology

### Materials

**Figure 1:**
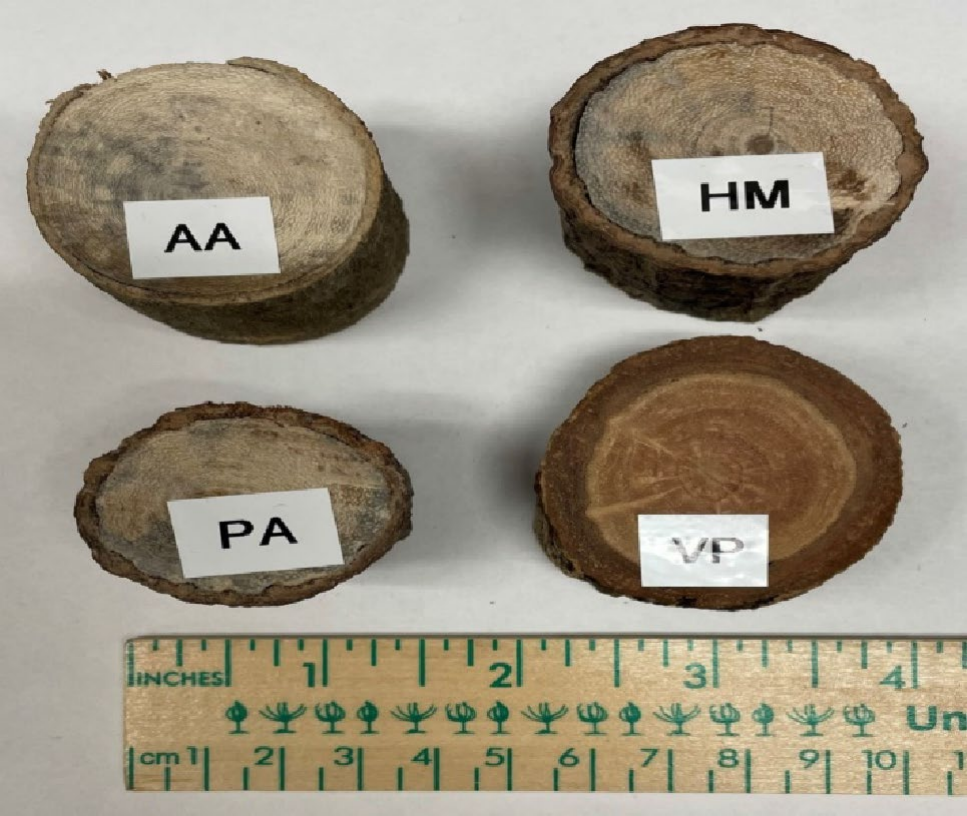
Photograph of AA, HM, PA ad VP wood stem cross-section.

Four Nigerian tree species (*Prosopis africana* (PA), *Harungana madascariensces* (HM), *Vitellaria paradoxa* (VP) and *Afzelia africana* (AA)), were collected from a farm site clearance in Ajase-ipo, Kwara state. A photograph of the four wood stem cross-sections is shown in Fig. 1. Charcoal was produced from stems and branches (1–2 m in length) containing bark stacked in an earth mound kiln. The stacked wood was covered with grass and sand. Small vent holes were made in the kiln to promote smooth, gentle burning. The earth mound was lit, and combustion took place for 11–48 h.

### Proximate analysis

The woody plant stems (wood containing bark) and charcoal materials (Fig. 1) were ground in a Thomas Wiley laboratory mill Model 4 and screened to < 0.5 mm and stored in Ziploc bags. Ash content was measured in duplicate at 600 °C for 16 h in a muffle furnace in accordance with ASTM D1102-84, and moisture content (MC) was measured using an HB 43-S Mettler Toledo moisture analyzer. Fixed carbon (FC) and volatile matter (VM) were measured in duplicate at 950 °C for 7 min in a muffle furnace following ASTM E870-82. The CE 440 elemental analyzer was used to perform an elemental analysis to determine the amounts of C, H, and N. Oxygen was determined using the difference. A Parr oxygen bomb calorimeter model 1341 was used to determine the higher heating value (HHV) of biomass samples in triplicate, according to ASTM D5865-04.

### Compositional analysis

Wood biomass composition was determined (extractives, lignin, and carbohydrate)^10^. The biomass (~ 4 g) was Soxhlet extracted with dichloromethane (CH_2_Cl_2_) for 16 h, in duplicate, and the extractives content determined gravimetrically after concentrating to dryness according to ASTM D1108-96. The Klason and acid soluble lignin were determined on extractived raw biomass (200 mg, in duplicate) according to ASTM D1106 and TAPPI technique T-222, respectively^11^. The lignin concentration was measured by a two-step acid hydrolysis (72%, 2 mL for 60 min at 30 °C, diluted to 4% H_2_SO_4_, and autoclaved for 30 min at 120 °C) and the yield was gravimetrically measured after oven drying. The acid soluble lignin concentration of the acid hydrolysate filtrate (250 mL) was measured at 205 nm (ε = 110 L/gcm) using a Thermoelectron Genesys 50 UV–VIS spectrophotometer (Hanover Park, IL, USA). According to ASTM E1758-01, a high pressure liquid chromatograph (HPLC) was used to analyse the neutral carbohydrates^12^. To the acid hydrolysate (5 mL), inositol (0.5 mg/mL, 1 mL) was introduced as an internal standard mixed, neutralized with lead carbonate, centrifuged, deionized in a column incorporating Amberlite IR-120 H^+^ (1 mL) and Amberlite IRA35 OH^−^ (1 mL) resins, and filtered through a 0.45 µm filter. Sugars were measured in duplicate by HPLC at 90 °C on elution with water (0.5 mL/min) utilizing two Rezex RPM columns in series (7.8 mm × 30 cm, Phenomenex, Torrance, CA) and fitted to a refractive index detector (Waters model 2414, Milford, Massachusetts, USA). Data were collected, and the analysis was performed using the N2000 chromatography application. The computation of sugar content incorporated a hydrolysis loss factor and an anhydro-sugar adjustment.

### Thermogravimetric analysis

Thermogravimetric analysis (Perkin Elmer TGA-7, Massachusetts, USA) with dry air purge (30 mL/min) was performed under isothermal and non-isothermal conditions in duplicate. The non-isothermal behavior of charcoal samples (4 mg) was examined from 30 to 1000 °C at a constant heating rate of 5, 10, and 20 °C/min. Isothermal conditions in which the temperature was ramped from 30 °C to the specified temperature of 350, 500, or 650 °C at 20 °C/min and then held isothermally for 60 min. Pyris v12 software was used to analyze the data. There was good repeatability between replicates.

### Kinetics methods

According to the conversion rate, the thermo-kinetics of solid-state matter can be expressed as follows^13^:

1 $$\frac{d\alpha}{dt}=kf(\alpha )$$

2 $$\alpha =\frac{{m}_{o}-{m}_{t}}{{m}_{o}-{m}_{f}}$$

where $\frac{d\alpha }{dt}$ is the conversion rate,$t$ is the reaction time,$\alpha$ is the conversion degree,${m}_{o}$ is the initial mass of the charcoal sample, ${m}_{t}$ is the mass of the charcoal sample at time t, ${m}_{f}$ is the final mass of the charcoal sample, $k$ is the constant of the reaction rate based on the Arrhenius law and can be calculated as follows:

3 $$k=A.exp\left(\frac{-{E}_{a}}{RT}\right)$$

where $A$ is the pre-exponential factor $({s}^{-1})$, $E$ is apparent activation energy $\left(kJ/mol\right)$

$R$ is the universal gas constant $\left(8.314 J/mol.K\right)$, $T$ is the absolute temperature $(K)$.

For the non-isothermal combustion experiment, the linear heating rate ($\beta$) is defined as follows:

4 $$\beta =\frac{dT}{dt}$$

Combining Eq. 1 to 4, the new function obtained is expressed thus:

5 $$\underset{0}{\overset{\alpha }{\int }}\frac{dx}{f(x)}=g\left(\alpha \right)=\frac{A}{\beta }\underset{{T}_{0}}{\overset{T}{\int }}exp\left(\frac{-{E}_{a}}{RT}\right).dT$$

where $g\left(\alpha \right)$ is the integral function of conversion.

### Model-free method

The model-free methods rely on evaluating the Arrhenius parameters without the need to determine the reaction order^13^. The methods are valid to analyze both isothermal and non-isothermal combustion. This involves a series of experiments performed at different heating rates. The two prevalent model-free methods, the Flynn–Wall–Ozawa (FWO) were used to calculate the apparent activation energy of the charcoal samples. The FWO method is a popular model-free method for determining the kinetic parameters of solid fuels^14^; the governing expression of the method is given by Eq. (6):

6 $$ln\beta =ln\frac{A.{E}_{a}}{R.g\left(\alpha \right)}-5.335-\frac{1.0516.{E}_{a}}{RT} $$

From the equation above, the regression lines of ln(β) versus 1/T, based on the same conversion at different temperature heating rates, give the activation energy obtained from the slope.

Upon obtaining the activation energy for the charcoal devolatization, the pre-exponential factor, *A*, was estimated using the equation below:

7 $$A=\frac{\beta.{E}_{a}.exp\left({E}_{a}/{R{T}_{p}}\right)}{R{{T}_{p}}^{2}}$$

### Model-fitting method

The Coats and Redfern (C–R) model-fitting approach was employed to determine the Ea and A. Within this method, the reaction model function (Table 1) is typically obtained using the Ea acquired from the model-free method. According to the Taylor series approach, the original equation obtained by Coats and Redfern for obtaining kinetic parameters can be expressed as follows:

**Table 1:** Reaction model^54^.

S/N	Reaction models	$f(\alpha )$	$g(\alpha )$
Nucleation models			
1	Power Law	$4{\alpha }^{3/4}$	${\alpha }^{1/4}$
2	Power Law	$3{\alpha }^{2/3}$	${\alpha }^{1/3}$
3	Power Law	$2{\alpha }^{1/2}$	${\alpha }^{1/2}$
4	Avrami-Erofeev	$4\left(1-\alpha \right){\left[-ln\left(1-\alpha \right)\right]}^{3/4}$	${\left[-ln\left(1-\alpha \right)\right]}^{1/4}$
5	Avrami-Erofeev	$3\left(1-\alpha \right){\left[-ln\left(1-\alpha \right)\right]}^{2/3}$	${\left[-ln\left(1-\alpha \right)\right]}^{1/3}$
6	Avrami-Erofeev	$2\left(1-\alpha \right){\left[-ln\left(1-\alpha \right)\right]}^{1/2}$	${\left[-ln\left(1-\alpha \right)\right]}^{1/2}$
Diffusion models			
7	One dimensional diffusion	$\left(1/2\right)\left(\alpha -1\right)$	$\alpha$
8	Diffusion control (janders)	$2{\left(1-\alpha \right)}^{2/3}.{\left[1-{\left(1-\alpha \right)}^{1/3}\right]}^{-1}$	${\left[1-{\left(1-\alpha \right)}^{1/3}\right]}^{2}$
9	Diffusion control (crank)	$3/2{\left[{\left(1-\alpha \right)}^{-1/3}-1\right]}^{-1}$	$1-2/3\alpha -{\left(1-\alpha \right)}^{2/3}$
Reaction order and geometrical contraction models			
10	Mampel (first order)	$\left(1-\alpha \right)$	$-ln\left(1-\alpha \right)$
11	Second order	${\left(1-\alpha \right)}^{n}$	$\left[{\left(1-\alpha \right)}^{-1}-1\right]$
12	Contracting cylinder	$2{\left(1-\alpha \right)}^{1/2}$	$1-{\left(1-\alpha \right)}^{1/2}$
13	Contracting sphere	$3{\left(1-\alpha \right)}^{2/3}$	$1-{\left(1-\alpha \right)}^{1/3}$

8 $$g\left(\alpha \right)=\frac{AR{{T}_{p}}^{2}}{ \beta .{E}_{a}}.\left(1-\frac{2RT}{ {E}_{a}}\right).exp\left(-\frac{{E}_{a}}{R{T}_{p}}\right)=\frac{A}{\beta }\underset{{T}_{0}}{\overset{T}{\int }}exp\left(\frac{-{E}_{a}}{RT}\right).dT$$

The revised and simplified form of this equation is then written thus:

9 $$ln\left[\frac{g\left(\alpha \right)}{{T}^{2}}\right]=ln\left(\frac{A.R}{\beta .{E}_{a}}\right)-\left(\frac{{E}_{a}}{RT}\right)$$

Fitting experimental data to Eq. 9 then becomes possible, especially after considering certain assumptions. One of these assumptions is that combustion analysis acknowledges that $\left\{\left(1-\frac{2RT}{{E}_{a}}\right)\approx 1\right\}$, thus producing a linear equation. The line relationship between $y=ln\left[\frac{g\left(\alpha \right)}{{T}^{2}}\right]$ and $x=\left(\frac{1}{T}\right)$ is clear with the slope $\left(-\frac{{E}_{a}}{R}\right)$ and y-intercept $ln\left(\frac{A.R}{\beta .{E}_{a}}\right)$, which are used to determine the kinetic parameters ${E}_{a}$ , respectively.

### Vibrational spectroscopy

Fourier transform infra-red (FTIR) spectra of biomass samples (in triplicate) were obtained using a Thermo-Nicolet iS5 spectrometer (Madison WI, USA) with a ZnSe attenuated total reflection (iD5-ATR) accessory. For the biochar samples, a Ge iD5-ATR accessory was employed. Omnic v9.3 software was used to correct the baseline and average the FTIR spectra (Thermo-Nicolet). Lignin syringyl to guaiacyl ratios (S/G) were determined from the relative band heights of 1460 cm^−1^/1512 cm^−1^^15^. Cellulose crystallinity was estimated using the cellulose total crystallinity index (TCI) from the normalized relative band heights of 1370 cm^−1^/2920 cm^−117^.

Raman spectra of charcoal samples (4 replicates) were recorded on an Alpha 300R Raman microscope (Witec, Ulm, Germany) at 532 nm excitation, 0.5 s acquisition time, and 10 scans per replicate. The spectra were averaged, baseline corrected, and the 1360 cm^−1^ and 1600 cm^−1^ bands were fitted to determine peak intensity using IGOR Pro v8 (Wavemetrics, Portland, OR, USA). The ratio of D (disordered, 1360 cm^−1^)/G (graphitic, 1600 cm^−1^) band intensities (I_D_/I_G_) was used to calculate an estimation of disordered carbon in the charcoal^16^.

### X-ray diffraction (XRD)

XRD analysis on biomass samples was performed using a Siemens D5000 diffractometer with a Cu-Kα radiation (λ = 0.154 nm) from 2θ = 4 to 40° at 0.05° steps. The diffractogram was baseline corrected, and the crystallinity index (CI) of cellulose in the wood was determined using peak a height method (Eq. 1).

10 $$CI \left(\%\right)=\left(\frac{{I}_{200}-{I}_{am}}{{I}_{200}}\right) \times 100$$

where, I_200_ and I_am_ are respectively the intensity of the main crystalline at (200) and amorphous^17^.

### Permission

The Study complies with local and national guidelines and regulations.

## Results and discussion

### Physico-chemical characterization of biomass

The utilization of biomass materials as source for energy-related applications is typically accompanied by physicochemical characterization since it provides crucial information about the feedstock's physical, chemical, and thermal properties as well as its structural profile^18^. In fact, for predictive purposes, several studies have established functional relationships between the higher heating value (HHV) of biomass and its physicochemical characteristics, namely Volatile matter (VM) or Fixed carbon (FC), ash, carbon (C), Nitrogen (N), and hydrogen (H)^19,20^. An essential quality to qualify a fuel that provides information on fuel breakdown is the combination of elemental analysis, compositional analysis, and calorific value. The ground woody biomass samples of PA, HM, VP, and AA were characterized for proximate analysis (Table 2).

**Table 2:** Calorific value, surface area, proximate and elemental analysis of woody biomass and charcoal samples.

Samples name	Raw				Charcoal			
	PA	HM	VP	AA	PA	HM	VP	AA
Ash (%)	1.24^d^	0.99^c^	3.03^b^	3.21^a^	2.6^c^	1.6^d^	4.7^a^	3.1^b^
FC (%)	19.9^c^	20.8^b^	23.6^a^	19.5^d^	77.8^a^	67.6^c^	57.5^d^	74.7^b^
VM (%)	78.9^a^	78.2^b^	73.4^d^	77.3^c^	19.6^d^	30.9^b^	37.8^a^	22.2^c^
FC:VM ratio	0.25	0.27	0.32	0.25	3.97	2.19	1.52	3.37
Calorific value (MJ/kg)	20.5^c^	21.25^a^	20.6^b^	20.5^c^	29.8^b^	28.6^c^	26.1^d^	30.2^a^
Surface area (m^2^ g^−1^)					67.0^c^	73.7^a^	0.66^d^	70.92^b^
C (%)	54.09^b^	55.95^a^	55.95^a^	52.83^c^	83.76^a^	78.95^c^	70.06^d^	83.17^b^
H (%)	8.1	7.0	6.7	6.1				
N (%)	0.56^c^	0.27^d^	0.58^b^	0.86^a^	0.70^b^	0.60^c^	0.73^a^	0.72^a^

Superscript letters represent significant difference between samples at a *p* = 0.05.

Of the woody biomass samples, AA had the highest ash content at 3.21%, while HM had the lowest at 0.99% (Table 2). The literature ash contents for PA and AA branches were 1.94% and 2.50%^21^respectively. PA had about a third of the ash content reported by Abah at 3.93%^22^. The ash content for VP was 3% which was higher than that reported by Ayeni et al. (2013) at 2%.

The PA sample had the highest VM content (78.9%), while the VP sample had the lowest (73.4%). The FC values for the samples ranged between 73% (VP) and 79% (PA). These values are in line with the literature for woody biomass^24^. As a measure of the characteristics of biomass combustion, the FC/VM, also known as the fuel ratio, should be < 1.5^25^. The FC/VM values obtained ranged from 0.25 to 0.32, which indicate that these woody biomass samples are good for combustion. The HHV for the woody biomass samples ranged between 20.5 and 21.3 MJ/kg and is in the range of biomass (13–28 MJ/kg)^26^. PA had a HHV of 20.5 MJ/kg and was comparable to those reported at 19.8 MJ/kg and at 18.9 MJ/kg^21,27^. AA had a lower HHV (20.5 MJ/kg) than that documented at 21.3 MJ/kg^21^.

Elemental analysis was performed on the woody biomass samples (Table 2). Nitrogen contents were between 0.27 and 0.86%, Carbon contents were between 53 and 56%, and Hydrogen contents between 6.1 and 8.1%. These values were in agreement with various lignocellulosic biomass materials^24^.

The CH_2_Cl_2_ extractives content for the 4 woody biomass samples was between 0.6 and 2.8% and in the range (0.3–4.5%) for tropical woods^28^. VP was shown to have a higher extractives content (2.8%) than reported by Ayeni et al. (2013) at 1.9%, and this may be due to acetone being used as a solvent.

Lignin is known to be an important contributor to the char content of pyrolyzed biomass and therefore a good indicator of potential charcoal yield^29^. HM was shown to have the highest total lignin content, at 37% (Table 3), which remains within the acceptable range for certain woody biomass^1^. AA has the lowest total lignin content of 28%, while PA and VP have 34% and 36%, respectively. VP lignin content was 17% higher than that reported by Ayeni et al. (2013). The presence of a considerable amount of bark (see Fig. 1) in the VP sample may contribute to a higher measured lignin content. No compositional data was available, even after an extensive literature search, for AA, HM, and PA wood materials.

**Table 3:** Compositional (extractives, lignin and carbohydrate) analysis for the woody biomass samples.

Compositional analysis	AA	HM	PA	VP
CH_2_Cl_2_ extract (%)	1.15 ± 0.02	1.57 ± 0.04	0.63 ± 0.05	2.84 ± 0.05
Xylan (%)	9.78 ± 0.48	7.77 ± 0.13	7.56 ± 0.13	4.79 ± 0.24
Glucan (%)	34.88 ± 1.18	34.01 ± 1.61	34.93 ± 0.92	27.61 ± 1.44
Galactan (%)	2.19 ± 0.19	1.40 ± 0.02	1.41 ± 0.10	2.30 ± 0.24
Arabinan (%)	0.29 ± 0.10	0.36 ± 0.06	0.52 ± 0.03	1.82 ± 0.10
Mannan (%)	1.11 ± 0.01	0.87 ± 0.06	1.78 ± 0.09	0.98 ± 0.01
Total neutral carbohydrates (%)	48.2 ± 1.6	44.4 ± 1.9	46.2 ± 1.0	37.5 ± 1.8
Klason lignin (%)	26.1 ± 0.05	34.8 ± 0.40	31.6 ± 0.1	34.0 ± 0.1
Acid soluble lignin (%)	2.53 ± 0.01	2.44 ± 0.04	2.66 ± 0.02	1.70 ± 0.01
Total lignin (%)	28.6 ± 0.02	37.3 ± 0.4	34.3 ± 0.1	35.7 ± 0.2

Carbohydrate composition (mainly cellulose and xylan) contributes to the structural properties of wood and therefore to its importance^30^. The main neutral sugars in the woody biomass samples were glucan or cellulose (28–35%) and xylan (4.8–9.8%) together with decreasing amounts of galactan, mannan, and arabinan (Table 3). VP was shown to have low glucan (28%) and hemicellulose (10%) values in comparison to the literature at 45 and 20%, respectively (Ayeni et al. 2013). The presence of a considerable amount of bark (see Fig. 1) in the sample is likely to contribute to a lower carbohydrate content.

**Figure 2:**
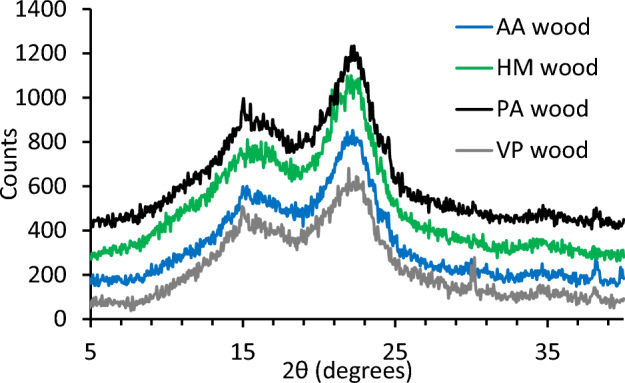
X-ray diffractograms of AA, HM, PA and VP woody biomass samples.

XRD analysis was performed on the woody biomass to determine the degree of cellulose crystallinity as CI. The tensile strength of wood is associated with crystalline cellulose^31^. The X-ray diffractograms (Fig. 2) were corrected for baseline and CI determined using the height method^17^. Calculated CI values for PA, HM, VP, and AA woody biomass samples after removing the amorphous (lignin and hemicellulose) components were 59%, 56%, 51%, and 55%, respectively. These CI values by XRD were comparable to those found for various wood species (43–56%)^32^.

**Figure 3:**
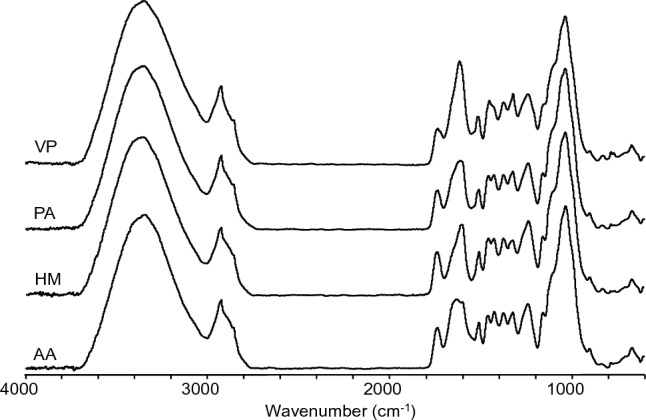
FTIR spectra of woody biomass samples AA, HM, PA and VP.

FTIR spectroscopy was employed to examine the functional groups of the woody biomass polymers (lignin, cellulose, and hemicellulose). Figure 3 shows the FTIR spectra of AA, HM, PA, and VP woody biomass. The strong band at 1034 cm^−1^ was assigned to glycosidic bonds (C–O–C) in polysaccharides^33^. Bands at 1740 cm^−1^ (C=O stretching of esters and acids), 1375 cm^−1^ (C–H bending), and 898 cm^−1^ (C–H deformation) were also assigned to polysaccharides^33^. Bands for lignin were observed at 1235 cm^−1^ (C=O, C–O, C–C bending in G units), 1325 cm^−1^ (S units ring breathing), 1460 cm^−1^ (C–H bending), 1515 cm^−1^ (C=C stretching of aromatic skeleton), and 1630 cm^−1^ (C=C stretching of aromatic skeleton)^15^. Cellulose TCI values in the woody biomass samples were determined from FTIR spectra. TCI values for AA, HM, PA and VP were 0.81, 0.84, 0.75, and 0.80, respectively. These results were higher than the TCI values determined for other woods (0.4–0.7). Lignin S/G ratios were also determined by FTIR spectroscopy and were 1.2 for AA and 1.3 for HM, PA, and VP. These S/G ratio values were in agreement (1.1–1.2) with those reported for hardwoods using the same method.

### Physico-chemical characterization of charcoal

The woody biomass limbs were converted into charcoal in an earth mound kiln using traditional methods for up to 2 days^35^. The VM content for the 4 charcoal samples ranged between 19.6 and 37.8%. The VM content significantly decreased as a result of burning wood. For example, the VM of PA wood decreased from 78.9 to 19.6% for charcoal (Table 2). The mean difference (mean difference = 49.3) between the pair of VM of wood and charcoal from the 4 tree species was found to be significant ((*p* ≤ 0.05), according to the pair assessment results. This fits well with findings from previous literature and is typical of lignocellulosic biomass products^36^. The VM decrease is a result of the dehydration and decarboxylation reactions during the thermal treatment of the biomass, a process known as devolatilization^30^. Comparing the study’s samples as presented in Tables 2 and 4, the proximate components of the raw and charcoal samples were significantly different (*p* ≤ 0.05) within the group. When compared to the studied charcoal samples from a study on metallurgical coke and non-coking coals^37^, each wood charcoal produced has more FC content, a lower ash content, and a lower VM content than non-coking coal. Thus, wood charcoal is suitable for the thermo-chemical conversion of biomass for heat generation.

**Table 4:** Result of pairwise t-test of parameters in raw and charcoal samples.

Paired samples test									
		Paired differences					t	*Df*	Sig. (2-tailed)
		Mean	Std. Deviation	Std. error mean	95% confidence interval of the difference				
					Lower	Upper			
Pair 1	ASHR—Ash	− 0.88333	0.72700	0.20987	− 1.34525	− 0.42142	− 4.209	11	0.001
Pair 2	FCR – FC	− 33.89833	28.92273	8.34927	− 52.27496	− 15.52171	− 4.060	11	0.002
Pair 3	VMR—VM	49.31833	9.38700	2.70979	43.35412	55.28255	18.200	11	0.000
Pair 4	CR—C	− 24.28000	6.82613	1.97053	− 28.61712	− 19.94288	− 12.322	11	0.000
Pair 5	NR—N	− 0.12083	0.17526	0.05059	− 0.23219	− 0.00948	− 2.388	11	0.036
Pair 6	CVR—CV	− 7.96917	1.73741	0.50155	− 9.07307	− 6.86527	− 15.889	11	0.000

The ash content of the charcoals was relatively low, between 1.6% and 4.7%. The results of the study's maximum ash content value of 4.7% for VP still remain within the specified limit of 5% for tropical wood^25^. The ash content of VP could be attributed to its high bark content (Fig. 1).

The data on the surface area of charcoal indicates the extent of carbonization (or reaction temperature) that has occurred during processing^38^. The surface areas of the charcoals were between 0.66 m^2^ g^−1^ (for VP) and 73.7 m^2^ g^−1^ (for HM). These surface areas were in the range (1–700 m^2^ g^−1^) for biomass chars^38^.

The HHV for the 4 charcoal samples (Table 2) were between 26.1 MJ/Kg and 30.2 MJ/Kg and consistent with the literature^39^. The charcoal sample's HHV values were in the following order: AA > PA > HM > VP. The charcoal calorific values compare well to a study on the combustion quality analysis of briquettes, which found that all of the materials met the minimum standards for a solid fuel rather than coal^19^. The caloric value reveals the quality of biomass, as its caloric value determines its energy contents^4^.

Elemental analyses of the 4 charcoal samples are given in Table 2. For wood charcoal samples, the C and N contents were AA: 83.2% and 0.72%, VP: 70.1% and 0.73%, HM: 79.0% and 0.60%, and PA: 83.8% and 0.70%, respectively. The carbonization of the wood significantly increased the C content of the charcoal to 70–84%; as expected, the N content was low at < 1%. The elemental composition results are in agreement with the literature and imply that if employed for energy application purposes, this would result in a rapid decline in nitrogen oxide emissions^1^.

**Figure 4:**
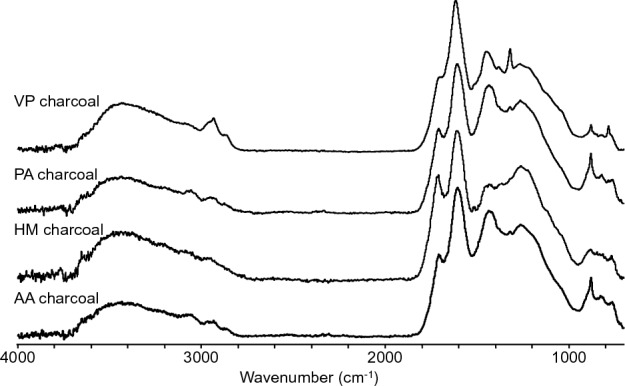
FTIR spectra of AA, HM, PA and VP charcoals.

The 4 charcoal samples were characterized by FTIR spectroscopy (Fig. 4). The spectra show a clear reduction in the O–H stretching band around 3500 cm^−1^, due to dehydration reactions as compared to the original wood. The charcoal samples show aliphatic 2860–3000 cm^−1^ and aromatic (3060 cm^−1^) C–H stretching bands^38^. A carbonyl band (1700 cm^−1^) was also observed in the charcoal samples. The charcoal spectra showed a strong aromatic ring stretching bands at 1610 cm^−1^ and 1440 cm^−1^ together with a smaller band at 1510 cm^−1^^40^. A band at 1323 cm^−1^ was assigned to aliphatic CH_2_ deformation^40^. Two sharp bands at 882 cm^−1^ and 793 cm^−1^ were assigned to substituted aromatic structures ^43^. These charcoal samples have bands that have been observed in other studies on charcoals^38,40,41^.

**Figure 5:**
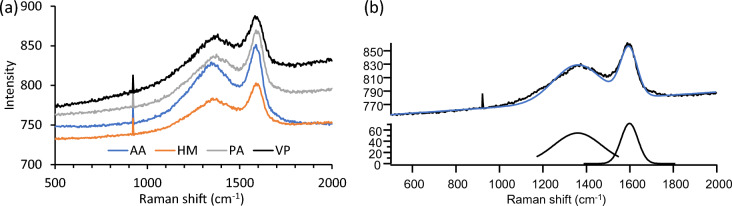
(**a**) Raman spectra for AA, HM, PA and VP charcoals and (**b**) band fitted Raman spectrum of PA charcoal.

Raman spectroscopy was applied to obtain information on the structure of C in the charcoal samples^42^. The Raman spectra for AA, HM, PA, and VP charcoals are shown in Fig. 5a. The proportion of disordered (amorphous) C and graphitic C can be determined by quantitatively analysing the relative Raman band heights (I_d_/I_g_) at about 1350 cm^−1^ (for disordered C) to 1600 cm^−1^ (graphitic C) after band fitting^43^. Figure 5b shows the band fitting applied to the Raman spectrum of PA charcoal. The I_d_/I_g_ ratio for AA charcoal was 0.9 and for HM, PA, and VP charcoals was 0.8. These I_d_/I_g_ values were in the range for biochars from lignocellulosic materials^16,44,45^.

## Thermo-analytical characterization of charcoal

**Figure 6:**
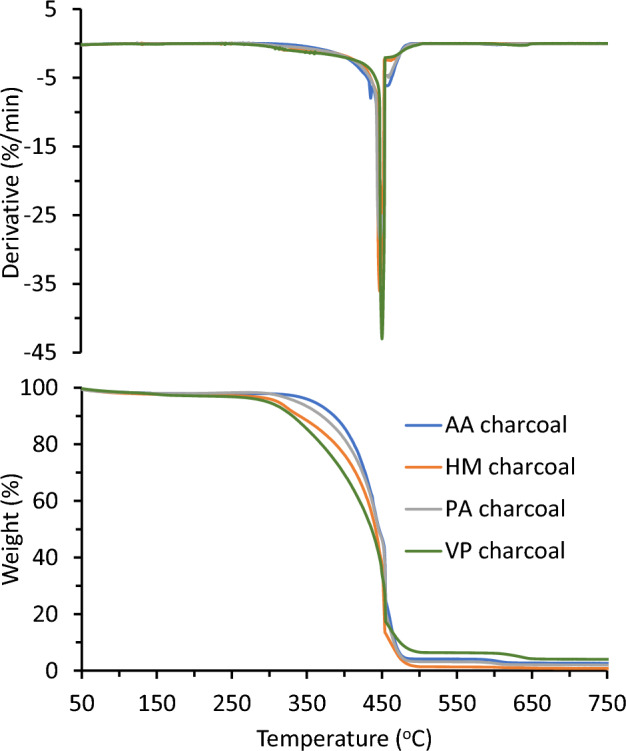
TGA (bottom) and DTG (top) thermograms of AA, HM, PA and VP charcoal samples in air at 5 °C/min.

**Dynamic thermogravimetric.** For information on the decomposition mechanisms in components of biomass samples, TGA and its first derivative (DTG) thermograms are useful^45^. TGA thermograms are also beneficial in devolatilization kinetics research, as are the qualitative data generated that can help in evaluating trends in close proximity to compositional analyses^46^. The TGA data that is obtained can be used for modelling, development, and optimisation in industrial applications. The TGA and DTG thermograms in air (combustion) were used to define the DTG peak, ignition, and burnout temperatures for the charcoal sample. Figure 6 displays the TGA and DTG thermograms for the 4 charcoal samples at 5 °C/min heating rate in air. The lowest temperature at which fuel spontaneously burns without the external influence of ignition is known as the ignition temperature, and the hottest temperature at which a fuel sample burns almost wholly through is known as the burnout temperature. Figure 6 depicts the thermal decomposition curves that were found at various heating rates. This type of degradation is typical of the majority of lignocellulosic biomass^47^. The moisture evaporation and loosening of several low-molar-mass organics are indicated by the DTG peaks. The broad DTG peaks that develop.

between 431 and 451 °C were predominantly caused by the breakdown of the carbohydrate portion. The majority of the mass loss at this point is consistent with the results for the carbohydrate content in Table 3. Since lignin has been demonstrated to breakdown over a greater temperature range, the slope > 650 °C reveals lignin degradation. Figure 6 further demonstrates the importance of heating rates on the thermal decomposition process, showing that an increase in the heating rate causes the DTG peaks and the beginning of decomposition to migrate toward higher temperature values while the thermal profiles remain the same. The thermal decomposition patterns supported the temperature dependence of the reaction rate and the fact that the decomposition mechanism is independent of the heating rate under the experimental conditions used, where the height of the derivative peaks increases with increasing heating rate, based on knowledge of reaction kinetics^48^. The ignition temperature, DTG peak, and burnout temperature for PA charcoal at a heating rate of 5 °C/min (Table 5) were 380 °C, 446 °C, and 486 °C, respectively. For the charcoal sample, the temperatures are as follows: AA = 386 °C, 451 °C, and 491 °C; VP = 380 °C, 436 °C, and 480 °C; and HM = 390 °C, 431 °C, and 520 °C. Similar patterns were seen for charcoal samples AA, VP, HM, and PA at 10 °C/min and 20 °C/min heating rates. Depending on the heating rate, cellulose exhibits a distinct first derivative maximum peak around 380 °C, which corresponds to a sharp decrease in weight. This is consistent with a finding made about cellulose decomposition in the literature^49^.

**Table 5:** TGA data showing Ignition, Burnout temperature and DTG peak for charcoal samples at 5 °C/min.

Charcoal sample	Ignition temperature (°C)	DTG peak (°C)	Burnout temperature (°C)
PA	380	446	486
AA	386	451	491
HM	390	431	520
VP	380	436	480

Decomposition begins with the loss of moisture and maybe some extractives, as observed in Fig. 6. Between 430 and 520 °C, the structures (disordered and graphitic carbons) of the charcoal samples were destroyed^47^. The DTG curves for the charcoal samples, as shown in Fig. 6, displayed three breakdown regimes and are similar to those observed for *Parthenium hysterophorus*^50^.

### Isothermal thermogravimetric

**Figure 7:**
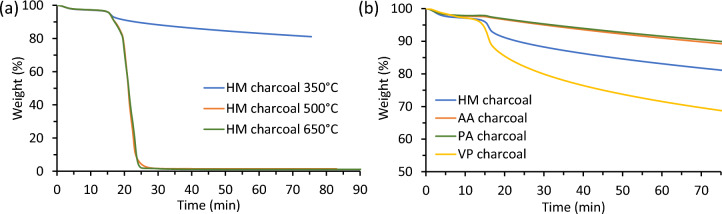
Isothermal thermograms of (**a**) HM charcoal samples at 350 °C, 500 °C and 650 °C and (**b**) AA, HM, PA and VP charcoals at 350 °C.

Isothermal TGA experiments were conducted by initially ramping at 20 °C/min to the specified temperatures of 350 °C, 500 °C, and 650 °C and held for 60 min for charcoal (thermally treated) samples. These experiments were performed to estimate the mass loss during combustion. Figure 7 shows isothermal thermograms of (a) HM charcoal at 350 °C, 500 °C and 650 °C and (b) AA, HM, PA and VP charcoal at 350 °C.

For the isothermal runs, the first 20 min is the ramp-up period during which the majority of moisture evaporation occurs, and this is an important step for comparison on a dry basis. Then the samples are heated isothermally for 60 min at the selected temperature. For creating a stove and performing a cost analyses for charcoal, the samples suddenly decreased in weight once reaching either 350 or 500 °C (Fig. 7a). The rationale is that lignin will lose the least weight loss while the hemicellulose is the most reactive^18^.

### Kinetic modeling for charcoal devolatization

To reduce the error of the hypothesis model, model-free methods were applied to obtain comparatively accurate kinetic parameters; subsequently, the model-free and model-fitting methods were combined to determine the optimal reaction model. According to the TG analysis, the intense combustion reaction mainly occurred in the second stage of the three stages. The first stage of mass loss was associated with dehydration and slow gas absorption. A small DTG peak was also observed, mainly due to moisture evaporation, while in the second stage, the charcoal samples experienced a substantial mass loss, this caused rapid and intense decomposition and oxidation of the sample. The major causes of the extensive mass loss could be closely associated with solid-state water evaporation, decomposition of organic matter, and combustion of volatile matter and fixed carbon. The third reaction stage was highly associated with char gasification and the dissociation of strong bonds. Thus, the TG/ DTG experimental data were used to determine the kinetic scheme of the charcoal combustion.

### Model-free method

**Figure 8:**
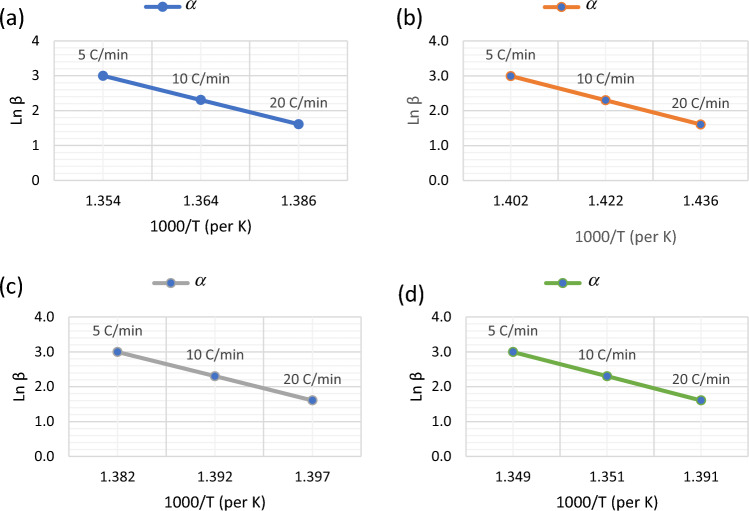
FWO Energy slope plots for (**a**) AA, (**b**) HM, (**c**) PA, and (**d**) VP.

In order to contribute to a deeper comprehension role of various heating rates (β = 5, 10, 20 °C min^−1^) on the thermal degradation of the charcoal samples, the apparent activation energy (*E*_*a*_) and pre-exponential factor (A) based on the same conversion at different temperature heating rates (Table 6), gives the activation energy obtained from the slope at several degrees of conversion were calculated by applying FWO isoconversional methods. From the Eq. (6), the regression lines of ln(β) versus 1/T, based on the same conversion at different temperature heating rates, which are represented by inclined vertical lines, reflect a linear dependence (Fig. 8 a,b,c and d for different charcoal samples). The calculated values of the *E*_*a*_ and frequency factor (min^−1^), from the slope and intercept, are given in Table 6. These isoconversional plots are parallel straight lines, which indicate a complex weight loss process with several mechanisms. The model-free method showed good results by describing an evolution of the apparent activation energy with the degree of conversion (α). Figure 8a,b,c and d show the slope for the straight lines obtained above for the four charcoal samples used in predicting the *E*_*a*_ of the various samples. After which, the constant and gradient for all the samples were achieved by solving the simultaneous equations obtained from all the graphs.

**Table 6:** Kinetic parameters of different charcoal samples.

Charcoal samples	Apparent activation Energy $(Ea$)$\left(kJ/mol\right)$	Pre-exponential factor $(A) ({min}^{-1})$
AA	548	2.88 × ${10}^{39}$
VP	274	$2.54\times {10}^{19}$
PA	548	$5.57\times {10}^{39}$
HM		$1.18\times {10}^{20}$

### Model-fitting technique

The *Ea* and A values for charcoal in air were calculated using the CR model (Table 7). These were derived from the slope of plots of ln(g T2) against 1 /T for various heating rates using Eq. (5). The reaction order, *n*, in the integral chemical reaction model could be either a positive or negative integer. The *E*_*a*_ for the second-order reaction model varies between 110 and 180 kJ/mol. The diffusional model exhibits the highest average *E*_*a*_ value of 155 kJ/mol for the thermal conditions, which is similar to the *E*_*a*_ of 170 kJ/mol from the integral model-free procedures for second stage. This suggests that diffusion was crucial to the degradation of the charcoal samples under study at this time. Solid-state reactions often take place between either the crystal lattices or the molecules penetrating the lattices, and this has been explained where the mobility of reactant constituents depends on lattice defects^51^.

**Table 7:** CR model results from Reaction and Diffusion Models for charcoal samples.

AA	Reaction Models	$ln\left(\frac{g(\alpha )}{{T}^{2}}\right)$	$ln\left(\frac{g(\alpha )}{{T}^{2}}\right)$	$-\frac{{E}_{a}}{R}$	$ln\left(\frac{A.R}{\beta .{E}_{a}}\right)$	${E}_{a}$
Diffusion Models		${350 }$°C	${500 }$°C		$\beta =10$	$kJ/mol$
1	1D diffusion	− 14.99	− 13.37	− 5188.94	− 6.66	43.12
2	Diff Control (J)	− 19.14	− 12.59	− 21,020.6	14.6	174.68
3	Diff Control (C)	− 19.25	− 14.86	− 14,095.96	3.37	117.14
Reaction order & geometrical contraction models						
4	Mampel (1st order)	− 14.93	− 12.32	− 8353.96	− 1.51	69.46
5	Second Order	− 14.86	− 10.71	− 13,316.16	6.51	110.66
6	Contracting Cylinder	− 15.65	− 13.61	− 6559.72	− 5.12	54.51
7	Contracting Sphere	− 16.05	− 13.83	− 7108.61	− 4.64	59.07

HM	Reaction Models	$ln\left(\frac{g(\alpha )}{{T}^{2}}\right)$	$ln\left(\frac{g(\alpha )}{{T}^{2}}\right)$	$-\frac{{E}_{a}}{R}$	$ln\left(\frac{A.R}{\beta .{E}_{a}}\right)$	${E}_{a}$
Diffusion Models		${350 }$°C	${500 }$ °C		$\beta =10$	$kJ/mol$
1	1D diffusion	− 14.53	− 13.32	− 3881.73	− 8.30	32.26
2	Diff Control (J)	− 18.11	− 11.33	− 21,780.82	16.85	180.99
3	Diff Control (C)	− 18.30	− 14.60	− 11,872.43	0.76	98.66
Reaction order & geometrical contraction models						
4	Mampel (1st order)	− 14.43	− 11.94	− 7993.6	− 1.60	66.43
5	Second Order	− 14.32	− 9.41	− 15,764.86	10.99	131.01
6	Contracting Cylinder	− 15.17	− 13.45	− 5517.75	− 6.31	45.85
7	Contracting Sphere	− 15.56	− 13.62	− 6236.75	− 5.55	51.83

PA	Reaction Models	$ln\left(\frac{g(\alpha )}{{T}^{2}}\right)$	$ln\left(\frac{g(\alpha )}{{T}^{2}}\right)$	$-\frac{{E}_{a}}{R}$	$ln\left(\frac{A.R}{\beta .{E}_{a}}\right)$	${E}_{a}$
Diffusion Models		${350 }$°C	${500 }$°C		$\beta =10$	$kJ/mol$
1	1D diffusion	− 15.17	− 13.34	− 5876.22	− 5.74	48.81
2	Diff Control (J)	− 19.53	− 11.99	− 24,207.7	19.32	201.17
3	Diff Control (C)	− 19.63	− 14.72	− 15,764.51	5.68	131.00
Reaction order & geometrical contraction models						
4	Mampel (1st order)	− 15.12	− 12.13	− 9592.84	0.28	79.72
5	Second Order	− 15.07	− 10.12	− 15,872.24	10.41	131.90
6	Contracting Cylinder	− 15.84	− 13.52	− 7432.79	− 3.91	61.77
7	Contracting Sphere	− 16.24	− 13.72	− 8079.23	− 3.27	67.14

VP	Reaction Models	$ln\left(\frac{g(\alpha )}{{T}^{2}}\right)$	$ln\left(\frac{g(\alpha )}{{T}^{2}}\right)$	$-\frac{{E}_{a}}{R}$	$ln\left(\frac{A.R}{\beta .{E}_{a}}\right)$	${E}_{a}$
Diffusion Models		${350 }$°C	${500 }$°C		$\beta =10$	($kJ/mol$
1	1D diffusion	− 14.01	− 13.41	− 1934.69	− 10.9	16.07
2	Diff Control (J)	− 16.84	− 13.01	− 12,291.94	2.89	102.15
3	Diff Control (C)	− 17.18	− 14.99	− 7039.28	− 5.88	58.50
Reaction order & geometrical contraction models						
4	Mampel (1st order)	− 13.82	− 12.47	− 4351.42	− 6.84	36.16
5	Second Order	− 13.62	− 11.10	− 8089.02	− 0.64	67.22
6	Contracting Cylinder	− 14.61	− 13.68	− 2983.26	− 9.82	24.79
7	Contracting Sphere	− 14.98	− 13.92	− 3401.91	− 9.52	28.27

For this analysis, the three stages previously discovered under the model-free kinetic study were examined for the best match in the CR approach using the reaction models displayed in Table 1. According to some studies, choosing an appropriate reaction mechanism can be accomplished by contrasting the average value of *E*_*a*_ derived from various heating rates with that of a model-free technique like FWO^51,52^. They proposed that a likely mechanism was represented by the integral model with the closest *E*_*a*_ among the ones provided. This hypothesis was applied in this current study. Additionally, the values of *E*_*a*_ that were in the same order of magnitude as the model-free kinetic data were taken into account due to a few unrealistic values that were found for several models at various phases of decomposition. Only the data from the second stage meets this condition based on the information above and those given in Table 7.

Potential reaction mechanisms that control the thermal breakdown process in oxidative conditions were represented by the chemical reaction and diffusional models^53,54^. The diffusion model, where the mobility of the reactants controls the reaction rate, best describes solid-state processes involving gaseous products^55^. In solid-state reactions involving gaseous products, where the rate of the reaction was governed by the movement of reactants or products from the reaction interface or product layer, the diffusion and reaction order models were used in this investigation because they provided the best explanation. Since the reaction sequence, or the concentration, amount, or fractions of reactant(s), resemble those used in homogeneous kinetics, order-based models are the simplest models used. In these models, the reaction sequence, or the concentration, amount, or fraction of reactant(s) left, was raised to a specific power (integral or fractional). The geometrical contraction model presupposes that nucleation happens quickly on the crystal's surface and that the rate of degradation is governed by the reaction interface's progression towards the crystal's centre.

## Conclusion

The four Nigerian woody biomass samples, AA, HM, PA, and VP, were effectively analysed for composition, thermally decomposed products, and thermal decomposition kinetics. Since identification revealed higher levels of lignin, ash, and extractives in the woody biomass and generated charcoal samples, there were noticeable changes in the composition of these biomass samples. The TGA clearly displayed the thermal degradation process for the examined charcoal samples of lignocellulosic biomass fractions as well as the impact of different heating rates. Their potential usefulness for bioenergy applications was also shown by the data obtained.

It was discovered that the apparent activation energy, *E*_*a*_, varied for different charcoal samples and most likely due to compositional differences. Elemental analyses of the four charcoal samples showed carbonization of the wood significantly increased the Carbon content of the charcoal to 70–84%. Regarding thermal decomposition, the derivative weight loss shows a clear difference in the way derived charcoal decomposes in the thermal atmospheres investigated. The model-fitting approach predicts that chemical reactions and diffusional models play dominant roles in the heat degradation of the charcoal. Thus, it was determined that the heat degradation of the relevant biomass predominantly involved intricate, multi-step reaction processes. The researched charcoal samples have demonstrated significant potential for bioenergy applications. The development of the related thermochemical processes as well as the design and optimization of reactors/combustors would greatly benefit from the kinetic data.

## Data Availability

The datasets used and/or analysed during the current study available from the corresponding author on reasonable request.
